# Dermolipome: aspect per-opératoire

**DOI:** 10.11604/pamj.2019.34.21.18120

**Published:** 2019-09-10

**Authors:** Yasmine Chaoui Roqai, Ajhoun Yousra

**Affiliations:** 1 Service d'Ophtalmologie, Hôpital Militaire d'Instruction Mohammed V, Rabat, Maroc

**Keywords:** Dermolipome, tumeur conjonctivale bénigne, exérèse chirurgicale, Dermolipoma, benign conjunctival tumor, surgical resection

## Abstract

Dermolipoma is an ectopic skin formation with a greasy component which develops at the level of the fornix of the external canthus. Surgery for dermolipoma is not devoid of dangers. There is essentially a risk of causing damage to the right external muscle and levator muscle of the upper eyelid because there is no cleavage plane between the lesion and the adjacent normal tissues to which it adheres. The anterior surface of the dermolipoma is inseparable from the temporal bulbar conjunctiva. Its posterior surface is adjacent to the right lateralis muscle and can extend up to the levator Müller's muscle complex of the upper eyelid and toward the lachrymal gland. We report the case of a 56-year old patient presenting with a yellowish white lesion of the external canthus of the right eye. The patient underwent orbital scanner in order to exclude the possibility of orbital extension and to establish the relationship with the different ocular structures. Given the aesthetic discomfort, the patient underwent surgical resection of the orbital grease in front of the orbital rim. The main differential diagnoses included conjunctival cyst, complex choristoma and dermoid cyst. Anatomopathological examination confirmed the diagnosis of dermolipoma. The postoperative course was uneventful.

## Image en medicine

Le dermolipome est une formation cutanée ectopique avec une composante graisseuse qui se développe au niveau du fornix du canthus externe. La chirurgie du dermolipome n'est pas dénuée de dangers, il existe essentiellement un risque de léser le muscle droit externe et le muscle releveur de la paupière supérieure; car il n'existe pas de plan de clivage entre la lésion et les tissus normaux adjacents auxquels elle adhère. La surface antérieure du dermolipome est indissociable de la conjonctive bulbaire temporale. Sa face postérieure est adjacente au muscle droit latéral et peut s'étendre en haut vers le complexe Müller-releveur de la paupière supérieure et vers la glande lacrymale. Nous rapportons le cas d'un patient âgé de 56 ans consultant pour une lésion blanc jaunâtre du canthus externe de l'œil droit, le patient a réalisé un scanner orbitaire afin d'éliminer une extention orbitaire et préciser les rapports avec les différentes structures oculaires devant la gêne esthétique le patient a bénéficié d'une exérèse chirurgicale de la graisse orbitaire en avant du rebord orbitaire. Les principaux diagnostics différentiels sont le kyste conjonctival, le choristome complexe, et le kyste dermoïde. L'étude anatomo-pathologique a confirmé le diagnostic de dermolipome. Les suites post-opératoires étaient simples.

**Figure 1:**
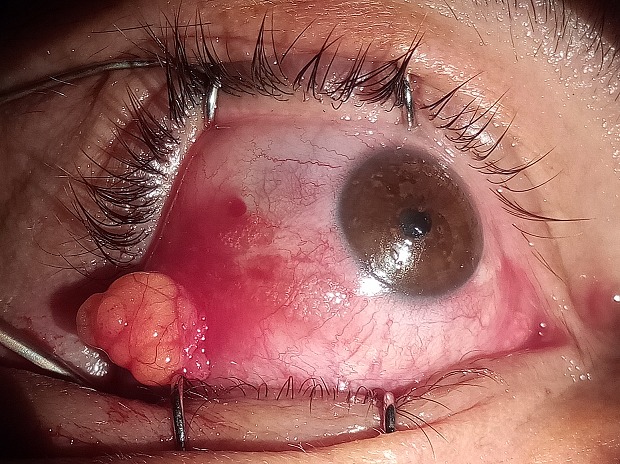
Aspect per-opératoire du dermolipome

